# Safety and Immunogenicity of the Heterologous 2-Dose Ad26.ZEBOV, MVA-BN-Filo Vaccine Regimen in Health Care Providers and Frontliners of the Democratic Republic of the Congo

**DOI:** 10.1093/infdis/jiad350

**Published:** 2023-08-24

**Authors:** Ynke Larivière, Irene Garcia-Fogeda, Trésor Zola Matuvanga, Bernard Isekah Osang'ir, Solange Milolo, Rachel Meta, Primo Kimbulu, Cynthia Robinson, Michael Katwere, Chelsea McLean, Niel Hens, Junior Matangila, Vivi Maketa, Patrick Mitashi, Hypolite Muhindo-Mavoko, Jean-Pierre Van geertruyden, Pierre Van Damme

**Affiliations:** Centre for the Evaluation of Vaccination, Vaccine and Infectious Disease Institute, University of Antwerp, Wilrijk; Global Health Institute, Department of Family Medicine and Population Health, University of Antwerp, Wilrijk; Centre for Health Economics Research and Modelling Infectious Diseases, Vaccine and Infectious Diseases Institute, University of Antwerp, Antwerp, Belgium; Centre for the Evaluation of Vaccination, Vaccine and Infectious Disease Institute, University of Antwerp, Wilrijk; Global Health Institute, Department of Family Medicine and Population Health, University of Antwerp, Wilrijk; Tropical Medicine Department, University of Kinshasa, Kinshasa, Democratic Republic of the Congo; Centre for the Evaluation of Vaccination, Vaccine and Infectious Disease Institute, University of Antwerp, Wilrijk; Global Health Institute, Department of Family Medicine and Population Health, University of Antwerp, Wilrijk; Tropical Medicine Department, University of Kinshasa, Kinshasa, Democratic Republic of the Congo; Tropical Medicine Department, University of Kinshasa, Kinshasa, Democratic Republic of the Congo; Tropical Medicine Department, University of Kinshasa, Kinshasa, Democratic Republic of the Congo; Janssen Vaccines & Prevention, Leiden, the Netherlands; Janssen Vaccines & Prevention, Leiden, the Netherlands; Janssen Vaccines & Prevention, Leiden, the Netherlands; Centre for Health Economics Research and Modelling Infectious Diseases, Vaccine and Infectious Diseases Institute, University of Antwerp, Antwerp, Belgium; Data Science Institute, Interuniversity Institute for Biostatistics and statistical Bioinformatics, UHasselt, Diepenbeek, Belgium; Tropical Medicine Department, University of Kinshasa, Kinshasa, Democratic Republic of the Congo; Tropical Medicine Department, University of Kinshasa, Kinshasa, Democratic Republic of the Congo; Tropical Medicine Department, University of Kinshasa, Kinshasa, Democratic Republic of the Congo; Tropical Medicine Department, University of Kinshasa, Kinshasa, Democratic Republic of the Congo; Global Health Institute, Department of Family Medicine and Population Health, University of Antwerp, Wilrijk; Centre for the Evaluation of Vaccination, Vaccine and Infectious Disease Institute, University of Antwerp, Wilrijk

**Keywords:** Ad26.ZEBOV, MVA-BN-Filo, health care providers and frontliners, safety and immunogenicity, ebola vaccine trial

## Abstract

**Background:**

In response to recent Ebola epidemics, vaccine development against the *Zaire ebolavirus* (EBOV) has been fast-tracked in the past decade. Health care providers and frontliners working in Ebola-endemic areas are at high risk of contracting and spreading the virus.

**Methods:**

This study assessed the safety and immunogenicity of the 2-dose heterologous Ad26.ZEBOV, MVA-BN-Filo vaccine regimen (administered at a 56-day interval) among 699 health care providers and frontliners taking part in a phase 2, monocentric, randomized vaccine trial in Boende, the Democratic Republic of Congo. The first participant was enrolled and vaccinated on 18 December 2019. Serious adverse events were collected up to 6 months after the last received dose. The EBOV glycoprotein FANG ELISA (Filovirus Animal Nonclinical Group enzyme-linked immunosorbent assay) was used to measure the immunoglobulin G–binding antibody response to the EBOV glycoprotein.

**Results:**

The vaccine regimen was well tolerated with no vaccine-related serious adverse events reported. Twenty-one days after the second dose, an EBOV glycoprotein–specific binding antibody response was observed in 95.2% of participants.

**Conclusions:**

The 2-dose vaccine regimen was well tolerated and led to a high antibody response among fully vaccinated health care providers and frontliners in Boende.

## Teaser

The administration of a heterologous Ad26.ZEBOV, MVA-BN-Filo vaccine regimen (56-day interval) in health care providers and frontliners from the Tshuapa province (Democratic Republic of the Congo) induced a robust Ebola virus glycoprotein–specific binding antibody response 21 days after the second dose.

Ebola virus disease (EVD) was discovered in 1976 and became known worldwide between 2013 and 2016 during the devastating West African epidemic. During this epidemic, EVD spread across multiple countries and infected >28 600 people with a 40% case fatality rate (CFR) [1]. The Democratic Republic of Congo (DRC) is the most afflicted country, with at least 15 outbreaks (CFRs ranging from 42% to 100%); 1 of which was an epidemic that led to 3470 cases and 2287 deaths (CFR, 66%) in the North Kivu, South Kivu, and Ituri provinces between 2018 and 2020 [1, 2]. When EVD epidemics occurred in unexpected locations (ie, 2013–2016 West Africa epidemic) or in politically unstable locations (ie, 2018–2020 Kivu and Ituri Ebola epidemic in the DRC), they have had a greater impact than previously expected possible [3, 4]. In response to these epidemics and the global health threat that EVD continues to pose, vaccine development against this deadly disease has been fast-tracked in the past decade [5].

Because of the unpredictability of when and where the next Ebola outbreak will occur [6] and considering the potential of vaccinating high-risk exposure groups such as health care providers and frontliners (hereafter, HCPs) [7, 8], the use of a vaccine that induces a durable and protective immune response is crucial. Janssen Vaccines & Prevention BV, together with Bavarian Nordic, developed the 2-dose heterologous Ad26.ZEBOV (Zabdeno) and MVA-BN-Filo (Mvabea) vaccine regimen. The Ad26.ZEBOV vaccine is a monovalent replication-incompetent adenoviral vector serotype 26 (Ad26) vaccine, encoding the full-length glycoprotein (GP) of the *Zaire ebolavirus* (EBOV) Mayinga variant [9]. The MVA-BN-Filo vaccine is a nonreplicating multivalent modified vaccinia Ankara (MVA) vaccine, encoding the EBOV Mayinga GP, the *Tai Forest ebolavirus* nucleoprotein, the *Sudan ebolavirus* Gulu GP, and the *Marburg virus* Musoke GP [9]. While it has not been possible to measure clinical efficacy with a classical clinical study, immunobridging analysis from nonhuman primates to humans supports the likelihood of protection [10], and the regimen was therefore granted marketing authorization by the European Medicines Agency in 2020 for use under “exceptional circumstances” as prophylactic vaccination in children and adults [11]. Preliminary studies for the Ad26.ZEBOV, MVA-BN-Filo heterologous vaccine regimen have shown that it is generally well tolerated and safe and leads to a durable immune response up to at least to 2 years after the initial vaccination [12–16].

The DRC's seventh Ebola outbreak took place in the Boende health district in 2014 [17]. Therefore, to protect HCPs in this Ebola-endemic region of the DRC, we performed a randomized vaccine trial whereby HCPs were first vaccinated with the Ad26.ZEBOV, MVA-BN-Filo vaccine regimen and then boosted with Ad26.ZEBOV either 1 or 2 years after the first dose (1:1 randomization) [18]. This article presents the safety and immunogenicity of the Ad26.ZEBOV vaccine as the first dose, followed by the MVA-BN-Filo vaccine as the second dose at a 56-day interval, in HCPs of the Boende health district of the Tshuapa province in the DRC.

## METHODS

### Study Participants

Participants had to be at least 18 years old and apparently healthy, pass a test of understanding (ie, a 10-question true/false questionnaire, for which 3 attempts were allowed to obtain a score of 9 or 10/10, assessing the participant's understanding of the trial and their consent), and have the means to be contacted. HCPs who were pregnant, breastfeeding, or planning to become pregnant within 3 months after the initial vaccination were excluded from enrollment. Further details on inclusion and exclusion criteria are provided by Larivière et al [18].

### Study Design and Procedures

**Figure 1.**
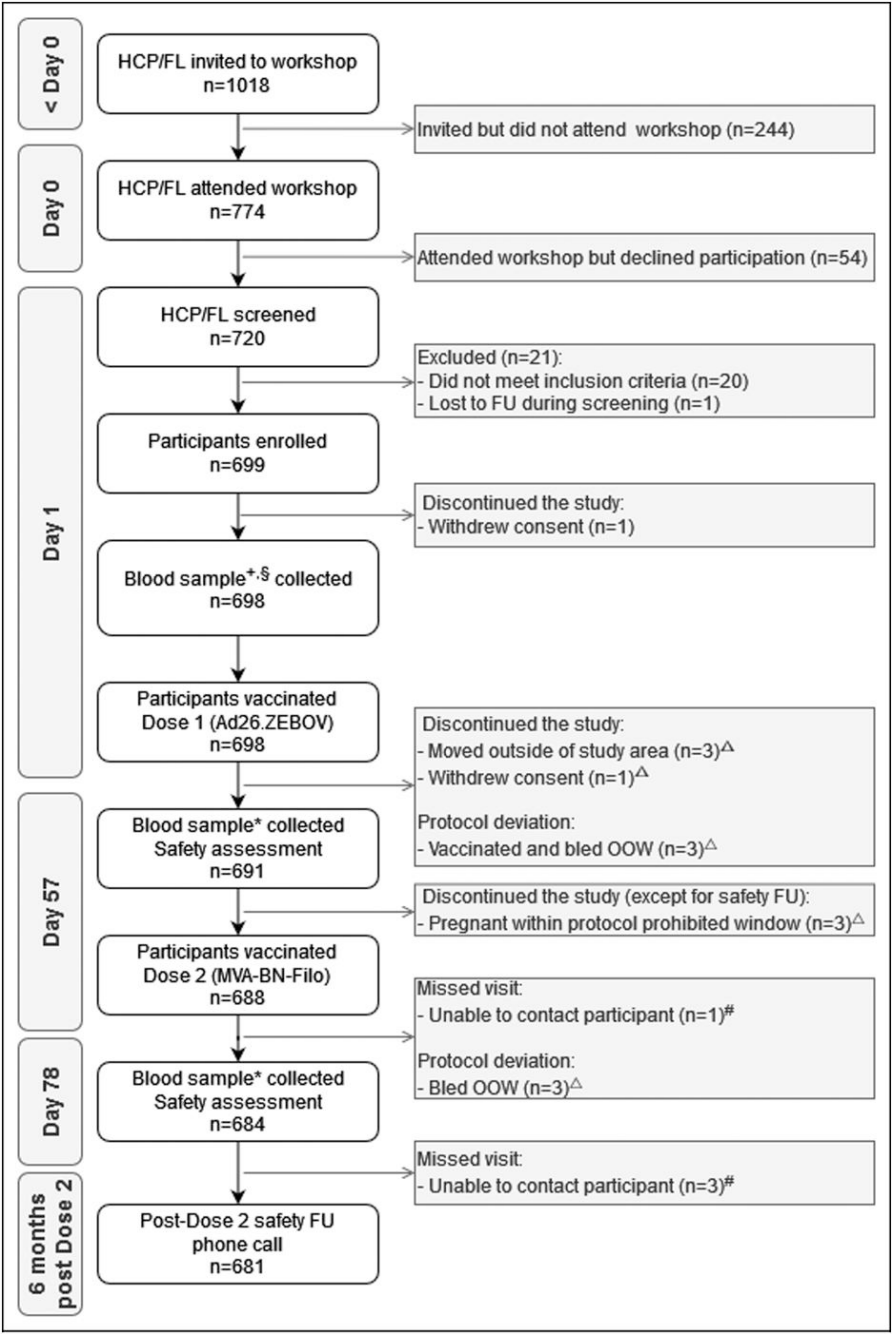
Study flowchart. Abbreviations: HCP = Health care providers; FL = frontliners; FU = Follow-up; OOW = Out of window; ^+^For baseline hematology, biochemistry and immunogenicity assessment; ^§^On Day 1, five samples were unable to be analyzed: four samples failed to meet acceptance criteria during multiple independent runs and one sample exceeded stability before a final result could be obtained; *For immunogenicity assessment; ^△^Part of full analysis set: all participants that received at least one dose of the heterologous Ad26.ZEBOV, MVA-BN-Filo vaccine regimen, irrespective of protocol deviations that occurred; ^#^Part of per protocol set: Ad26.ZEBOV, MVA-BN-Filo regimen received and at least one immonogenicity sample post vaccination and no protocol deviations with impact on immunogenicity.

Based on convenience sampling, enrollment targeted 700 participants starting in December 2019 for a vaccine trial with an open-label, monocentric, randomized design (Figure 1). On day 0, in maximum groups of 40 individuals, registered HCPs working in the Boende health district were invited to attend a workshop where the informed consent form was explained. If they were willing to participate in the study after attending the workshop, they were asked to return the next day for screening and consent (day 1). In case a HCP was illiterate, a literate third party not involved in the conduct of the study served as a witness to the consenting procedure and was asked to sign the informed consent form if the HCP agreed to participate. This article addresses the primary and one of the secondary objectives of the trial, which assessed the safety and immunogenicity of the heterologous 2-dose vaccine regimen in HCPs working in the Boende health district of the Tshuapa province in the DRC. Additionally, in a subset of participants, the exploratory objectives assessed the impact of the presence of baseline neutralizing antibodies against Ad26 and MVA vectors on the EBOV-specific immune response. Information on the clinical trial itself is available on www.clinicaltrials.gov (NCT04186000), and study procedures are explained in detail by Larivière et al [18]. The trial was approved by the National Ethics Committee of the Ministry of Health of the DRC (121/CNES/BN/PMMF/2019) and the Ethics Committee of the University Hospital of Antwerp/University of Antwerp (19/14/177).

#### Safety Assessment

Participants remained in observation for 30 minutes after the Ad26.ZEBOV vaccination on day 1 and the MVA-BN-Filo vaccination on day 57. The presence of any serious adverse events (SAEs)—related or unrelated to the investigational product (IP; Ad26.ZEBOV or MVA-BN-Filo) and as defined by the E2A clinical safety data management scientific guideline of the International Conference on Harmonisation [19]—was assessed up to 6 months after the last received dose.

#### Immunogenicity Assessment

Blood samples were collected from all participants to identify human anti–EBOV GP immunoglobulin G (IgG) antibody levels on day 1 (prevaccination, baseline immunogenicity assessment), day 57 (prevaccination, dose 1 immunogenicity assessment), and day 78 (dose 2 immunogenicity assessment).

All samples were analyzed at Q² Solutions Vaccine Testing Laboratory with the EBOV GP FANG ELISA (Filovirus Animal Nonclinical Group enzyme-linked immunosorbent assay) [20]. This validated assay was used to measure the IgG antibody concentrations against EBOV surface GP in the collected serum.

From the first 98 participants enrolled in the trial, additional serum was collected at baseline to assess the presence of neutralizing antibodies against the Ad26 and MVA vector backbone by using Ad26 and MVA virus backbone neutralizattion assays. The Ad26 virus neutralization assay (Ad26 VNA) was developed, qualified, and performed by Janssen Vaccines & Prevention BV, and the MVA virus neutralization assay (ie, human vaccinia plaque reduction neutralization assay) was developed, validated, and performed by Bavarian Nordic.

### Data Management and Analysis

#### Data Management

Data were collected in French on paper source documents and then transcribed into the electronic database (DFdiscover version 5.2.0). All data were reviewed by the principal investigator or delegated staff. Monitors of a clinical research organization performed source data verification. All open-field translations from French to English were certified.

#### Demographics and Safety Data Analysis

The full analysis set was used to analyze demographics, baseline characteristics, and safety data. This included all participants who received at least 1 dose of the heterologous vaccine regimen, irrespective of the protocol deviations that occurred. Descriptive statistics were used to present these data in number (%), mean (SD), or median (range). All safety data were coded with MedDRA coding (version 22.1) and presented with the MedDRA Preferred Term.

#### Immunogenicity Analysis

The immunogenicity analysis was conducted with the per-protocol set (PPS). This consisted of all participants who received both vaccinations and had at least 1 postvaccination immunogenicity result and no major protocol deviations with a consequence on immunogenicity. Anti–EBOV GP IgG geometric mean concentrations with 95% CIs were calculated for all available time points (days 1, 57, and 78). Participants were considered responders when they tested below or equal to the lower limit of quantification (LLOQ; ≤36.11 ELISA units [EU]/mL) at baseline and >2.5× LLOQ after baseline or had at least a 2.5-fold increase in antibodies after vaccination if they were already above the LLOQ at baseline. Except for calculation of the response rates, all values below or equal to the LLOQ (≤36.11 EU/mL) were imputed with half the value (18.06 EU/mL) to account for censoring in the parameter estimation. On a subset of participants, the Spearman correlation was assessed between preexisting neutralizing antibodies against the Ad26 and MVA vector and the anti–EBOV GP IgG antibody response before and after vaccination.

Statistical modeling was performed to investigate whether and how the following relate to differences in the mean antibody response (µ) and variability (σ): time in days between 2 collected blood samples, sex (male or female), age, previous vaccination with a third-generation smallpox vaccine (IMVAMUNE [also known as MVA-BN, JYNNEOS, and IMVANEX]; Bavarian Nordic A/S) against mpox (formerly monkeypox), and profession. Details on the methodology of this statistical model are available in the supplementary material.

All statistical analyses were performed in R (version 4.2.2); for statistical modeling, the *gamlss* package (version 5.4.3) was used.

## RESULTS

### Full Analysis Set

#### Demographic Characteristics

Enrollment began on 18 December 2019, and the post–dose 2 safety follow-up phone call visits ended on 23 October 2020. Data collected up to 23 October 2020 were used for analyses. Overall, 699 participants were enrolled (Figure 1). One participant withdrew consent before any study-related activity could be performed. Thus, the full analysis set consisted of 698 participants. All enrolled participants were Black and of African descent, and 76.5% were male (Table 1). The study population had a median age of 46.0 years, and the majority of the participants were community health workers (33.8%). Nurses and first aid workers were the second- and third-largest HCP groups, representing 25.9% and 25.4% of the participants, respectively. The majority of the participants worked in health centers (53.2%), for the Red Cross (25.4%), or in hospitals (12.0%). Out of 698 participants, 129 (18.5%) were vaccinated with a third-generation smallpox vaccine (IMVAMUNE) against mpox during a vaccine trial conducted in Boende in 2017 [21].

**Table 1.** Baseline Sociodemographic Characteristics: Full Analysis Set

Characteristic	No. (%)^a^
Sex	
Male	534 (76.5)
Female	164 (23.5)
Black	698 (100.0)
Age, y	
Mean (SD)	45.0 (12.0)
Median (range)	46.0 (19.0–75.0)
Profession	
Community health worker	236 (33.8)
Nurse	181 (25.9)
First aid worker	177 (25.4)
Hygienist	37 (5.3)
Midwife	30 (4.3)
Doctor	13 (1.9)
Health facility cleaner	10 (1.4)
Care giver	7 (1.0)
Laboratory technician	2 (0.3)
Pharmacist aid	2 (0.3)
Other	3 (0.4)
Work establishment	
Health center	371 (53.2)
Red Cross	177 (25.4)
Hospital	84 (12.0)
Health post	37 (5.3)
Health area	10 (1.4)
Provincial health department	9 (1.3)
Health zone	8 (1.2)
Health inspection	1 (0.1)
Staff member of the expanded program on immunization	1 (0.1)
Smallpox vaccination against mpox	
Yes	129 (18.5)
No	569 (81.5)

Health care providers and frontliners vaccinated with Ad26.ZEBOV, MVA-BN-Filo vaccine regimen in Boende, the Democratic Republic of the Congo, December 2019 (participants who received at least 1 study vaccine dose, N = 698).

^a^Data are presented as No. (%) unless indicated otherwise.

#### Safety Assessment

For the 698 participants who received at least 1 vaccination, no SAEs related to the IP were reported up to 6 months post–dose 2. In total, 31 SAEs unrelated to the IP were recorded among 20 participants. Of the 31 SAEs, 58.1% were considered severe, 35.5% moderate, and 6.5% mild (numbers add to 100.1% due to rounding). Overall, 77.4% of participants with (a) SAE(s) recovered or their SAE(s) resolved; 9.7% recovered/resolved with sequelae; and 3 died during the study—1 from HIV infection (diagnosis unknown at recruitment), 1 from dermohypodermitis, and 1 from ureterolithiasis and calculus bladder—accounting for 12.9% of SAEs. Further details on all reported SAEs that started between enrollment and 23 October 2020 are presented in Supplementary Table 1.

### Immunogenicity Assessment

#### Per-Protocol Analysis: PPS

Of the 688 participants who received 2 doses, 3 were excluded from PPS due to a protocol deviation with impact on immunogenicity. Therefore, the immunogenicity analyses consisted of 685 PPS participants. Five serum samples (0.7%), collected on day 1, were unable to be analyzed: 4 failed to meet acceptance criteria during multiple independent runs, and 1 exceeded stability before a final result could be obtained. For participants with missing baseline results, it was not possible to determine if they were responders.

**Figure 2.**
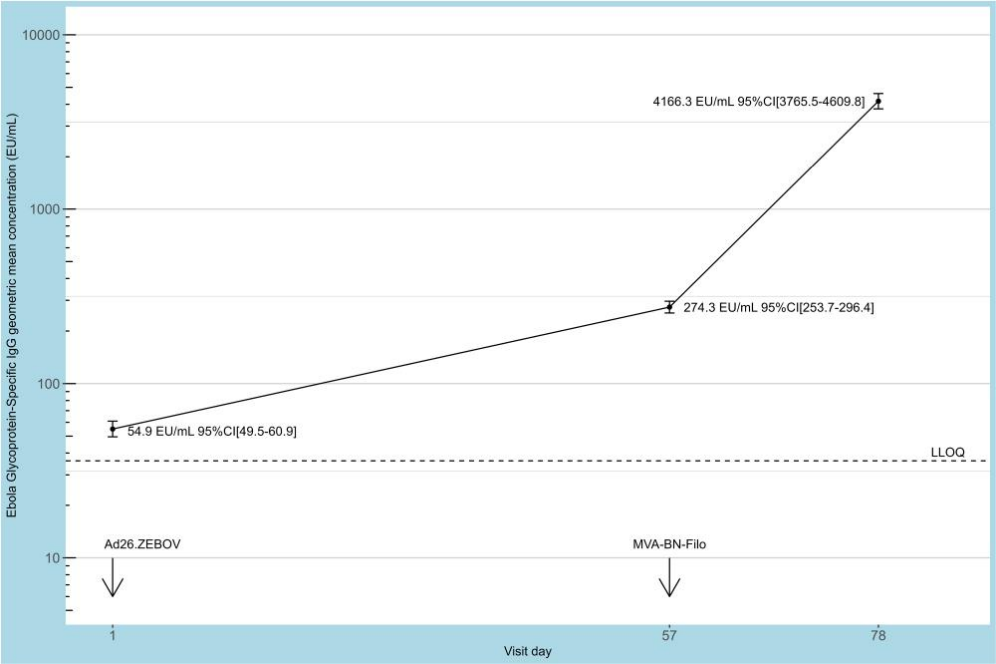
Geometric mean concentrations with 95% CIs of EBOV-specific binding antibodies. The lower limit of quantification is indicated by a dashed line (36.11 EU/mL). The Ad26.ZEBOV vaccine was administered at day 1 as the first dose, followed by the MVA-BN-Filo vaccine on day 57 (±7 days) as second dose at a 56-day interval. Blood samples were collected prior to the first dose as baseline, prior to the second dose on day 57 (±7 days), and at 21 days (day 78 ± 7 days) after the second dose to assess the humoral immune response after vaccination. EBOV, *Zaire ebolavirus*; EU, enzyme-linked immunosorbent assay unit; LLOQ, lower limit of quantification.

At baseline, participants had an anti-EBOV GP IgG geometric mean concentration of 54.8 EU/mL (95% CI, 49.4–60.8) (Figure 2), with 340 (49.6%) having antibody responses below or equal to the LLOQ. After dose 1, the geometric mean concentration increased to 274.3 EU/mL (95% CI, 253.8–296.4) at day 57 and 4166.3 EU/mL (95% CI, 3765.5–4609.8) 21 days after dose 2. This indicates a 5-fold increase in antibodies 56 days after administration of the first dose and a further 15-fold increase 21 days after administration of the second dose. After a single Ad26.ZEBOV vaccination (day 57 immune response), 431 participants (62.9%) were considered responders. Of the 679 participants for whom the immune response could be assessed at approximately 21 days after vaccination with the Ad26.ZEBOV, MVA-BN-Filo heterologous vaccine regimen (1 participant did not attend the day 78 visit), 652 (95.2%) were considered responders.

Finally, for a subset of 95 PPS participants, baseline Ad26- and MVA-specific seroprevalence rates of 93.7% and 70.5% were calculated, respectively. Negligible correlations were observed between Ad26-specific neutralizing antibodies at baseline and EBOV GP–specific binding antibodies 56 days post–dose 1 and 21 days post–dose 2 (Spearman correlation coefficients, −0.21 and −0.14), as well as between MVA-specific neutralizing antibodies at baseline and EBOV GP–specific binding antibodies 56 days post–dose 1 and 21 days post–dose 2 (Spearman correlation coefficients, −0.08 and −0.31). Based on these weak correlations (Supplementary Figure 1), there is no indication that the presence of the Ad26- and MVA-specific neutralizing antibodies had an impact on the vaccine-induced immune responses after vaccination.

#### Statistical Modeling

Statistical modeling indicates that there is a significant increase in the mean antibody response after each vaccination, with variability in the response declining by 59.8% and 71.3% for the day 57 and 78 visits, respectively, as compared with baseline (Table 2). This indicates an increase in homogeneity of the antibody response at each blood collection time point. While men started with a higher antibody response at baseline than women, a clear boost in antibody response was observed in men and women from day 57 until day 70, with women reaching a higher antibody response than men from day 70 onward (Supplementary Figure 2). For participants vaccinated against mpox, the EBOV GP–specific binding antibody response increased by 34% between the second and third visits. In contrast, for participants not vaccinated against mpox, a lower increase of 26% was observed between days 57 and 78. When assessing the profession, the estimated mean antibody response for first aid workers was 8% higher on average than for community health care workers (95% CI, 5%–20%). Finally, at baseline younger participants (quartile 1, age 36 years) had a 43% higher mean antibody response than older participants (quartile 3, age 54 years), and this difference persisted after vaccination (no significant change in variability over time for age).

**Table 2.** Mean Response and Variability Coefficients as Determined by the GAMLSS Model

Coefficient	Estimate	SE	*P* Value
µ: mean antibody response			
(Intercept)	4.363	0.095	<.001
Age	−0.025	0.001	<.001
Mpox received	0.494	0.169	.003
Profession			
First aid worker	0.089	0.039	.024
Nurse	−0.072	0.049	.143
Other	−0.075	0.055	.171
Sex: male	0.104	0.037	.005
Time in days	0.062	0.001	<.001
Mpox received × time in days (interaction)	−0.014	0.003	<.001
σ: variability			
(Intercept)	0.339	0.060	<.001
Time in days	−0.017	0.001	<.001
Sex: male	0.110	0.045	.014
Profession			
First aid worker	0.078	0.045	.081
Nurse	0.217	0.046	<.001
Other	0.205	0.059	<.001

Abbreviation: GAMLSS, generalized additive model for location, scale, and shape.

## DISCUSSION

Overall, the Ad26.ZEBOV, MVA-BN-Filo vaccine regimen (administered 56 days apart) was safe and led to a clear humoral immune response among study participants. In this study, no IP-related SAEs were observed up to 6 months after vaccination with the heterologous 2-dose vaccination series. One study previously paused vaccination in adults with the Ad26.ZEBOV, MVA-BN-Filo regimen after 2 neurologic SAEs (1 possibly related to the IP) were reported within a short interval at different stages of the vaccine regimen [22]. However, the study resumed when an external expert panel of neurologists did not raise any specific safety concerns [22]. Of the per-protocol vaccinated HCPs in our study, 95.2% were considered responders roughly 21 days after the second dose. This is a high response rate and similar to what was observed in previous studies assessing the EBOV GP–specific binding antibody response to this heterologous regimen among adult participants (ie, 98.0%–100.0% responder rates) [13, 15, 16, 22–26].

Evaluating vaccine efficacy against EBOV infection is extremely challenging due to the sporadic nature and unpredictable location of the next Ebola outbreak. The World Health Organization’s Strategic Advisory Group of Experts on Immunization currently recommends the single-dose rVSV-ZEBOV-GP (Ervebo) vaccine for use in high-risk populations during Ebola outbreaks [27]. This vaccine has shown 97.5%–100.0% clinical efficacy from day 10 after vaccination through ring vaccination in the DRC and Guinea [28, 29]. While ring vaccination during EVD outbreaks is recommended with the rVSV-ZEBOV-GP vaccine, in June 2021 the strategic advisory group’s recommendations were amended to include the vaccination of populations at lower risk of contracting EVD (eg, HCPs in neighboring regions to an outbreak) with the Ad26.ZEBOV, MVA-BN-Filo vaccine regimen [27]. Using immunobridging of EBOV GP–binding antibody responses between nonhuman primates and humans, Bockstal et al calculated a mean predicted survival probability of 53.4% (95% CI, 36.7%–67.4%) among humans vaccinated with the Ad26.ZEBOV, MVA-BN-Filo vaccine regimen [30]. Due to the strictness of the parameters under which the model was built, this is expected to be an underestimation of the actual vaccine efficacy in humans.

Responses to vaccination can vary for individuals depending on factors such as age and sex [31, 32]. Even though no formal statistical modeling of the immune response was initially foreseen for the current study, post hoc statistical modeling was able to provide new insights. For example, the model indicated that female and younger participants had a higher mean EBOV-specific antibody response after full vaccination, as compared with their male and older counterparts, respectively. Researchers have attributed differences in vaccine responses to (1) hormonal changes among male and female aging and their influence on the immune system and (2) the deterioration of adaptive immune responses with age [31–33]. However, interpretations of this model should be handled with caution, as a correlate of protection for EBOV-specific antibodies remains unknown [34]. Despite the observed differences in the model, 95.2% of participants were considered vaccine responders. Therefore, differences in EBOV-specific antibody response based on certain variables (eg, sex, profession, age) may be clinically irrelevant.

While the preexisting Ad26-specific neutralizing antibody response observed in this study was similar to responses observed by Ishola et al (93.7% vs 93.0%–94.0%, respectively), the preexisting MVA-specific neutralizing antibody response was considerably higher (70.5% vs 5.0%–17.0%) [15]. There are several possible explanations for the high MVA-specific reactivity at baseline. First, this could be attributed to the high number of participants (50 of 95) who were vaccinated against mpox with IMVAMUNE, a live modified vaccinia virus Ankara vaccine, in a vaccine trial conducted in the same study area in 2017 [21]. Second, smallpox vaccination, a live virus vaccinia vaccine, was part of the routine vaccination in the DRC until 1977 (when the DRC was officially declared smallpox-free), and sporadic vaccination continued until 1984 [35]. As the median age of the study population was 46.0 years, several participants would have been vaccinated against smallpox. Finally, the Tshuapa province, of which Boende is the capital, is considered a mpox-endemic area with an elevated incidence among HCPs as compared with the general public [21]. Cross-reactivity between local exposure to the monkeypox virus with the vaccinia virus could have occurred [36]. However, while the MVA-specific neutralizing antibody titer was considerably higher, only weak correlations were observed between the EBOV-specific binding antibodies and the MVA-specific neutralizing antibodies, indicating no apparent impact on the EBOV-specific binding antibody response.

This study has some limitations, such as the imbalance of male and female participants (76.5% and 23.5%, respectively) most likely due to socioeconomic and cultural factors within the local health care system. This imbalance was potentially enhanced through the exclusion of pregnant and breastfeeding women at enrollment, as is often the case during trials assessing a candidate IP [37]. As a second limitation, the HIV status of participants was mostly unknown, as this was not an exclusion criterion for the trial if the general condition of the participant was good and he or she was taking suppressive therapy. Only if participants disclosed their HIV status at screening or during the course of the trial was this information recorded. However, a previous study assessed the antibody response of the Ad26.ZEBOV, MVA-BN-Filo vaccine regimen in healthy adults and those who were HIV infected (well controlled by highly active antiretroviral therapy), and the authors found that the HIV status of participants did not have an apparent influence on the immune response as compared with healthy adults [24]. Finally, the data presented in this study were limited to a short immunogenicity assessment period after vaccination (up to 21 days after the second dose). Nevertheless, results show that the EBOV-specific immune response approximately 21 days after Ad26.ZEBOV and MVA-BN-Filo vaccination is high (ie, 95.2%) among vaccinated HCPs working in this Ebola-endemic area. Ultimately, the vaccination of this population therefore contributes to the epidemic preparedness within the Boende health district.

This study also has several strengths, including the high retention rate of participants and the vaccination of an at-risk population in an EBOV-endemic location. Epidemiologic modeling provides evidence that prophylactic vaccination of a small proportion of HCPs in an endemic at-risk location could significantly reduce Ebola incidence and associated mortality [38, 39]. As the 698 HCPs vaccinated in this study are a risk group working in an Ebola-endemic area, they may function as a sentinel demonstrating clinical efficacy if a new outbreak would occur in the region. Also, to the best of our knowledge, the current study is the first to assess the correlation between MVA-specific neutralizing antibodies at baseline and EBOV GP–specific binding antibodies after dose 1 and dose 2. Finally, by analyzing the data through a statistical model, more insights into variables affecting the immune response were achieved.

By recognizing the unpredictability of the next outbreak location and the potential of the prophylactic use of the Ad26.ZEBOV, MVA-BN-Filo vaccine regimen, preventative vaccination of HCPs working in Ebola-endemic areas could help prevent drastic consequences of the next Ebola outbreak. To ensure that prophylactic vaccination is useful, a durable immune response is crucial after vaccination. The next step within our study is to determine the durability of these vaccines among the HCP population, as well as their potential to induce an immune memory response through the administration of an Ad26.ZEBOV booster dose 1 or 2 years after vaccination.

## Supplementary Data

Supplementary materials are available at *The Journal of Infectious Diseases* online. Consisting of data provided by the authors to benefit the reader, the posted materials are not copyedited and are the sole responsibility of the authors, so questions or comments should be addressed to the corresponding author.

## Supplementary Material

jiad350_Supplementary_Data

## Notes

***Author contributions.*** Y. L. performed all analyses included in the manuscript (except statistical modeling) and wrote the manuscript. I. G.-F. performed analyses for the statistical model under the guidance of N. H. B. O. performed data management for the trial at the sponsor level. P. K. performed data management for the trial at the principal investigator level. H. M.-M. was principal investigator of the trial, with P. M. and J. M. as co–principal investigators. The University of Antwerp (P. V. D., J.-P. V. G.) was the sponsor of the trial. Y. L. was project manager at the sponsor level. V. M. was project manager at the investigator level. T. M. Z. was the study site coordinator. S. M. and R. M. were assistant site coordinators. S. M. was in charge of safety and cold chain and investigational product management. C. R., M. K., and C. M. were involved in trial discussions at the investigational product level. All authors reviewed and contributed to the final manuscript.

***Acknowledgments.*** We acknowledge the hard work and dedication of the local nurses, medical doctors, and laboratory technicians, working in sync with medical doctors, laboratory technicians, and data managers from the University of Kinshasa. This study would not have been possible without the entire EBL2007 study group (supplementary material). A special thank-you to Dr Emmanuel Esanga, Mme Maguy, Dr Solange Milolo, Dr Rachel Meta, Primo Kimbulu, and Pitchou Kasongo for their investment in this trial and for ensuring that it continued to run smoothly for its entire duration. We also acknowledge, with gratitude, the support provided by the Africa Contract Research Organization, with a special thank-you to Victorine Owira, and by DFNet Research through the development and maintenance of the study database, with a special thank-you to Yvonne Hong and Karolyn Scott. Finally and most important, we thank all the health care providers and frontliners who took time out of their busy schedules to take part in this trial as participants.

***Financial support.*** The EBOVAC3 project has received funding from the Innovative Medicines Initiative 2 (IMI2) Joint Undertaking under grant agreement 800176. This joint undertaking receives support from the European Union’s Horizon 2020 research and innovation program, the European Federation of Pharmaceutical Industries and Associations, and the Coalition for Epidemic Preparedness Innovations . For this trial, the contract research organization and part of the FANG ELISA analyses are funded by the Coalition for Epidemic Preparedness Innovations. All other trial activities are funded by the IMI2 Joint Undertaking grant. All vaccines and neutralizing antibody–level analyses against Ad26 at the first visit are provided by Janssen Vaccines & Prevention BV.
