# Supplementary material for: Safety and Immunogenicity of the Heterologous 2-Dose Ad26.ZEBOV, MVA-BN-Filo Vaccine Regimen in Health Care Providers and Frontliners of the Democratic Republic of the Congo
Source: J Infect Dis. 2023 Aug 24;229(4):1068–76. doi: 10.1093/infdis/jiad350 (PMC11011182; doi:10.1093/infdis/jiad350)
Supplement: jiad350_Supplementary_Data [file jiad350_supplementary_data.zip › renamed_45afd.docx]

Supplementary material

**Table of contents**

[1. EBL2007 study group 2](#_Toc140506626)

[2. Supplementary methods 5](#_Toc140506627)

[Methodology statistical model 5](#_Toc140506628)

[*3.* Supplementary results 7](#_Toc140506629)

[Table 1. Overview of SAEs starting before 23 October 2020 (FAS) of HCW and FL vaccinated with Ad26.ZEBOV, MVA-BN-Filo vaccine regimen in Boende, the Democratic Republic of the Congo 7](#_Toc140506630)

[Figure 1. Scatterplots and spearman correlations of pre-existing neutralizing antibody titres against the Ad26- and MVA-vector versus the anti-EBOV GP IgG antibody response before and after vaccination (Per protocol set 1) 9](#_Toc140506631)

[Figure 2. varying coefficient spline of time between blood samples for each level of the variable sex 11](#_Toc140506632)

# EBL2007 study group

List of contributors to the study over the four-year study duration period (2018-2022):

**Local study team in Boende**

Study managers: Hypolite Muhindo Mavoko, Junior Matangila Rika, Patrick Mitashi Mulopo, Vivi Maketa; Site coordinator: Emmanuel Esanga Longomo, Trésor Zola Matuvanga; Assistant site coordinators: Solange Milolo Tshilumba, Rachel Meta; Data managers: Pitchou Kasongo Bile, Daniel Kipasa Mambu, Primo Kimbulu Lumba; Study medical doctors: Rachel Meta, Michael Bojabwa Mondjo, Danoff Endbu Elunzi, Lazare Bakongo Isofefu, Lucien Nkoyi, Yves Tchuma Bisimwa, Jimmy Mpato Manga, Bienvenue Bolingo; Study nurses: Benedicte Liuba Balao, Rebecca Asieli Malaza, Jeanette Likinda, Guylain Mondje Bakongo, Sandra Mpia Bienga, Junior Mputu Ikomoli, Clarisse Ikuma Bampunga, Kanza Baye Nsase, Amba Boongo, Marguerite Mbenga Lolu; Study laboratory technicians and biologists: Elisabeth Mukundi Madinda, Patience Masinga Mbuku, Rodin Mukele Lungaba, Trésor Lipetsi Loyenga, Blandine Bokomo Belenge, Claudine Bakambo Luende; Cold chain and IP management: Solange Milolo Tshilumba, Emannual Esanga, Michael Bobjabwa Mondjo; Pharmacist: Francis Ngoy Kankienza; Safety management team: Rachel Meta, Yves Tchuma Bisimwa, Trésor Zola Matuvanga; Site financial coordinator: Maguy Issekitolo Fatuma Mpona; Electrician: Likali Bofuke; Study facility cleaners: Sorros, Lokuli Lokwa, Bofete Liweli; Study facility guards: Nicolas Boya Likuwa, Jean Bakalo Mpeti, Bokongola Ifambe, Daudin Lokuli

**Principal investigator team (University of Kinshasa)**

Principal investigator and co-principal investigators: Hypolite Muhindo Mavoko, Junior Matangila Rika, Patrick Mitashi Mulopo; Study coordinator: Vivi Maketa; Site coordinators: Tresor Zola Matuvanga, Solange Milolo Tshilumba; Data managers: Pitchou Kasongo Bile, Daniel Kipasa Mambu, Primo Kimbulu Lumba; Logistics coordinators: Rody Loshinga Masudi; Financial officer: Feza Gisèle Nabazungu; Principal administrator: Phanuel Katembo; Social scientists: Freddy Bikioli; assistant social scientist: Henri Kimina; Cold chain team Kinshasa: Japhet Kabalu Tshiongo, Daddy Mangungulu Bambi

**Sponsor team (University of Antwerp):**

Sponsor and co-sponsor: Pierre Van Damme, Jean-Pierre Van geertruyden; Personal assistant sponsor: Nele Brusselaers; Project managers: Bonome Nturo, Elke Stoppie, Ynke Larivière, Jessie De Bie, Gwen Lemey; Administrative and financial coordinators: Jan Vervoort, Gwen Lemey, Peter Vermeiren; Data managers: Swabra Nakato, Alfred Dusabimana, Ynke Larivière, Bernard Osang’ir; Statistician: Bernard Osang’ir; Medical reviewers: Kanchana Withanage, Katie Steenackers, Ilse De Coster, Marie-Annick Götze; Lead social scientists: Séverine Thys, Antea Paviotti; Social scientist: Maha Salloum; Modelling lead: Niel Hens; Modeller: Irene Garcia-Fogeda

**Janssen team**

Global program leader: Kim Offergeld, Annick Bessems; Global Trial Leader: Helga Pissens, Ines Martinez Vazquez, Adriana Hollestein; Global clinical trial assistant: Agnieszka Kwasniak, Kinga Mojzes;Disease management program leader: Paula Mc Kenna; Senior trial supply management specialist: Ellen Teunissen, Katrien Aerts; Trial supply manager: Megan Seels; Regulatory CTA submission manager: Jade Yee; Lead assosciate-submissions manager: Tomeka Harris, Guusje Hoogeveen; Medical leader: Cynthia Robinson; Global data manager: Regina In’t Veld, Ernesto Fernandez, Julia Chiang; Data delivery senior manager: Tinne De Cnodder; Project manager J&J global public health: Anneleen Vuchelen; Study responsible physician; Joachim Doua, Katwere Michael; Regional trial manager: Joel Nawatsi; Trial supply team lead: Nanou Van Gils; Local safety officer contact: Amani Ghadban, Damelya Medetbekova; Project lead clinical immunology: Griet Van Roey, Maaike Ligthart; Clinical supplies integrator: Tom Reijns, Max Grafe, Sohandra Randrasana; Statistical programming lead: Lee Armishaw, Meenakshi Behl; Statistical leader: Auguste Gaddah; Regulatory medical writer: Marleen Van Looveren; Quality assurance representative: Carolyn Artis

**Clinical Research Organization team (ACE Research)**

CEO ACE research: Odika Apollo, Victoria Tifft; Project Manager: Victorine Owira; Project manager: Sue Chase, Leslie Shupenko; In-country project manager: Andy Numbi, Dacquin Kasumba; Regulatory affairs: Amos Ndhere; Safety manager: Lucas Tina; Project specialist: Oliver Kipkemei; Clinical monitors: Jerry Liwono, Trésor Bodjick, Zakaria Gansane, James Okwach, Willy Mutangala, Simon Pierre Kisisa, Ken Awuondo; Quality assurance: Gonzaga Onyuka, Lillian Nambuchi, Penina Apudo

**Data management team (DFNet)**

President DFNet: Lisa Ondrejcek; Director Biometrics: Gavin Robertson; Clinical coding supervisor; Brian Postle; Data managers: Brian Postle, Yvonne Hong, Karolyn Scott; Statistical programmer: Jerad Post; Clinical coding lead: Khris Kline

# Supplementary methods

## Methodology statistical model

To achieve symmetry in the antibody response, values were log-transformed. To further account for the high number of participants with values below or equal to the LLOQ at baseline, a normal left-censored distribution was applied in the model. Due to the presence of censoring, non-linearity and individual variability in the data, several statistical models were explored, concluding that the assumption of a constant variability was not realistic. Therefore, a statistical Generalized Additive Model for Location, Scale and Shape (GAMLSS) was used. Variables that were assessed were: time in days between two collected blood samples, sex (male or female), age, previous vaccination with a third-generation smallpox vaccine (IMVAMUNE^®^ (also known as MVA-BN^®^, JYNNEOS^®^, and IMVANEX^®^), Bavarian Nordic A/S, Kvistgaard, Denmark) against mpox (formerly monkeypox), and profession. To obtain large enough categories per variable for the model, participants not working as community health workers, nurses or first aid workers, were grouped under the profession category “other”. In the mean response (µ) of the model, varying coefficient smoothing terms against time between blood sample collection for each level of the factor sex were fitted. In addition, random effects were used to account for the individual variability in the mean response. Parameter estimation in GAMLSS is performed using the method of maximum likelihood.

**The GAMLSS model:**

The full GAMLSS model can be defined as follows:

Y_i_ ~ D(µ_i_, σ_i_, ν_i_, τ_i_) independently for i=1, …, n

where D(·) is a parametric distribution, having up to four parameters µ, σ, ν, and τ representing the mean, variance, skewness, and kurtosis respectively. We define below how we modeled our parameters (µ, σ), where the function s() represents a variety of different effects: smooth terms and random effects. The functions g(k), k=1, …, 4 for the different parameters are of the following (linear) form:

g(µ) = β_0_ + β_i_*X_i_ + s_p_(X_i_)

g(σ) = α_0_ + α_i_*X_i_

g(ν) = ʎ_0_

g(τ) = θ_0_

# Supplementary results

## ***Table 1. Overview of SAEs starting before 23 October 2020 (FAS) of HCP vaccinated with Ad26.ZEBOV, MVA-BN-Filo vaccine regimen in Boende, the Democratic Republic of the Congo***

| **S**  **A**  **E** | **MeDra Preferred Term** | **Start date** | **End date** | **Toxicity grade** | **Relatedness to IP** | **Outcome** | **Congenital anomaly** | **Persistent disability** | **Hospitalization** | **Life-threatening** | **Other medically important event** |
| --- | --- | --- | --- | --- | --- | --- | --- | --- | --- | --- | --- |
| 1 | Ovarian cyst | 5-Feb-20 | 15-Feb-20 | Moderate | Not related to IP | Recovered/resolved | No | No | Yes | No | No |
| 2 | Uterine leiomyoma | 5-Feb-20 | 15-Feb-20 | Moderate | Not related to IP | Recovered/resolved | No | No | Yes | No | No |
| 3 | Enteritis | 11-Feb-20 | 15-Feb-20 | Moderate | Not related to IP | Recovered/resolved | No | No | Yes | No | No |
| 4 | Lower limb fracture | 12-Feb-20 | 18-Feb-20 | Moderate | Not related to IP | Recovered/resolved with sequelae | No | No | Yes | No | No |
| 5 | Skin ulcer | 18-Feb-20 | 18-Apr-20 | Severe | Not related to IP | Recovered/resolved | No | No | Yes | No | No |
| 6 | Cerebrovascular accident | 15-Mar-20 | 20-Mar-20 | Severe | Not related to IP | Recovered/resolved with sequelae | No | No | Yes | No | No |
| 7 | Abortion spontaneous | 30-Mar-20 | 31-Mar-20 | Moderate | Not related to IP | Recovered/resolved | No | No | No | No | Yes |
| 8 | Malaria | 30-Mar-20 | 2-Apr-20 | Severe | Not related to IP | Recovered/resolved | No | No | Yes | No | No |
| 9 | Typhoid fever | 30-Mar-20 | 2-Apr-20 | Moderate | Not related to IP | Recovered/resolved | No | No | Yes | No | No |
| 10 | HIV infection | 15-Apr-20 | 21-May-2021* | Severe | Not related to IP | Fatal | No | No | No | No | Yes |
| 11 | Dyspepsia | 15-Jun-20 | 20-Jun-20 | Mild | Not related to IP | Recovered/resolved | No | No | Yes | No | No |
| 12 | Asthenia | 16-Jun-20 | 21-Jun-20 | Severe | Not related to IP | Recovered/resolved | No | No | Yes | No | No |
| 13 | Pyrexia | 16-Jun-20 | 21-Jun-20 | Mild | Not related to IP | Recovered/resolved | No | No | Yes | No | No |
| 14 | Cerebrovascular accident | 25-Jun-20 | 20-Jul-20 | Severe | Not related to IP | Recovered/resolved with sequelae | No | No | Yes | No | No |
| 15 | Malaria | 1-Jul-20 | 6-Jul-20 | Severe | Not related to IP | Recovered/resolved | No | No | Yes | No | No |
| 16 | Pneumonia | 1-Jul-20 | 6-Jul-20 | Moderate | Not related to IP | Recovered/resolved | No | No | Yes | No | No |
| 17 | Abdominal strangulated hernia | 14-Jul-20 | 19-Jul-20 | Moderate | Not related to IP | Recovered/resolved | No | No | Yes | No | No |
| 18 | Malaria | 23-Jul-20 | 26-Jul-20 | Severe | Not related to IP | Recovered/resolved | No | No | Yes | No | No |
| 19 | Typhoid fever | 23-Jul-20 | 26-Jul-20 | Moderate | Not related to IP | Recovered/resolved | No | No | Yes | No | No |
| 20 | Calculus bladder | 27-Jul-20 | 21-Aug-20 | Severe | Not related to IP | Recovered/resolved | No | No | Yes | No | No |
| 21 | Malaria | 2-Aug-20 | 6-Aug-20 | Severe | Not related to IP | Recovered/resolved | No | No | Yes | No | No |
| 22 | Dehydration | 2-Aug-20 | 6-Aug-20 | Severe | Not related to IP | Recovered/resolved | No | No | Yes | No | No |
| 23 | Dermo-hypodermitis | 13-Aug-20 | 20-Jan-2021* | Severe | Not related to IP | Fatal | No | Yes | No | No | No |
| 24 | Abdominal adhesions | 22-Aug-20 | 31-Aug-20 | Severe | Not related to IP | Recovered/resolved | No | No | Yes | No | No |
| 25 | Malaria | 29-Aug-20 | 11-Sep-20 | Severe | Not related to IP | Recovered/resolved | No | No | Yes | No | No |
| 26 | Typhoid fever | 29-Aug-20 | 11-Sep-20 | Severe | Not related to IP | Recovered/resolved | No | No | Yes | No | No |
| 27 | Dehydration | 29-Aug-20 | 11-Sep-20 | Severe | Not related to IP | Recovered/resolved | No | No | Yes | No | No |
| 28 | Abortion spontaneous | 7-Oct-20 | 7-Oct-20 | Moderate | Not related to IP | Recovered/resolved | No | No | No | No | Yes |
| 29 | Malaria | 7-Oct-20 | 12-Oct-20 | Moderate | Not related to IP | Recovered/resolved | No | No | No | No | Yes |
| 30 | Ureterolithiasis | 15-Oct-20 | 10-Nov-2020* | Severe | Not related to IP | Fatal | No | No | Yes | No | No |
| 31 | Calculus bladder | 15-Oct-20 | 10-Nov-2020* | Severe | Not related to IP | Fatal | No | No | Yes | No | No |
| * On October 23^rd^, 2020, four SAEs were still ongoing. However, end dates and outcomes for these SAEs are reported as they were available at the time of analysis. | | | | | | | | | | | |

## Figure 1. Scatterplots and spearman correlations of pre-existing neutralizing antibody titres against the Ad26- and MVA-vector versus the anti-EBOV GP IgG antibody response before and after vaccination (Per protocol set 1)


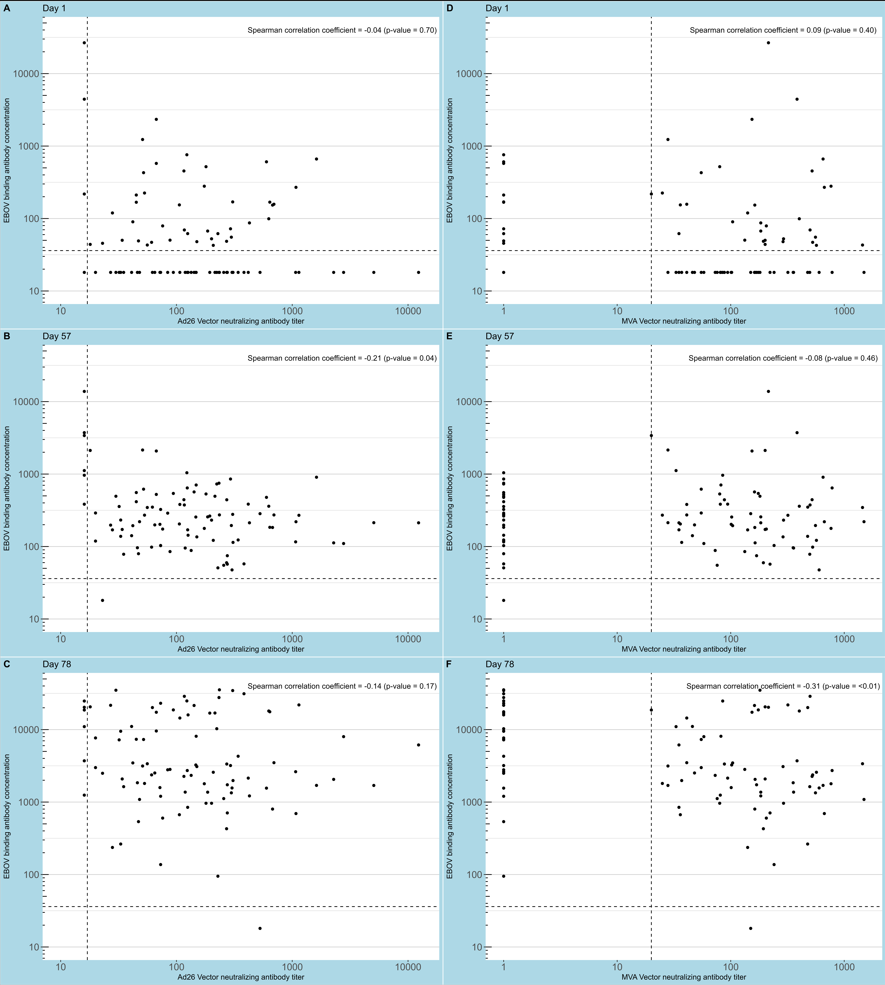


On the left, scatterplots between Ebola virus binding antibody concentrations and Ad26 neutralizing antbody titres are depicted with spearman correlation coefficients; on the right, scatterplots between Ebola virus binding antibody concentrations and MVA neutralizing antbody titres are depicted with spearman correlation coefficients; Panels A and D show the relation before vaccination (Day 1/baseline), panels B and E show the relation 56 days (±7 days) after vaccination with Ad26.ZEBOV (Day 57) and panels C and F present the relation 21 days (±7 days) after MVA-BN-Filo vaccination (Day 78); Horizontal dashed lines indicate the lower limit of quantification of the Ebola virus binding antibody concentrations and were set at 36.11 ELISA units/mL; Vertical dashed lines indicate the lower limit of quantification of Ad26- and MVA-vector antibody titres and were set at 17 and 20, respectively.

## Figure 2. varying coefficient spline of time between blood samples for each level of the variable sex


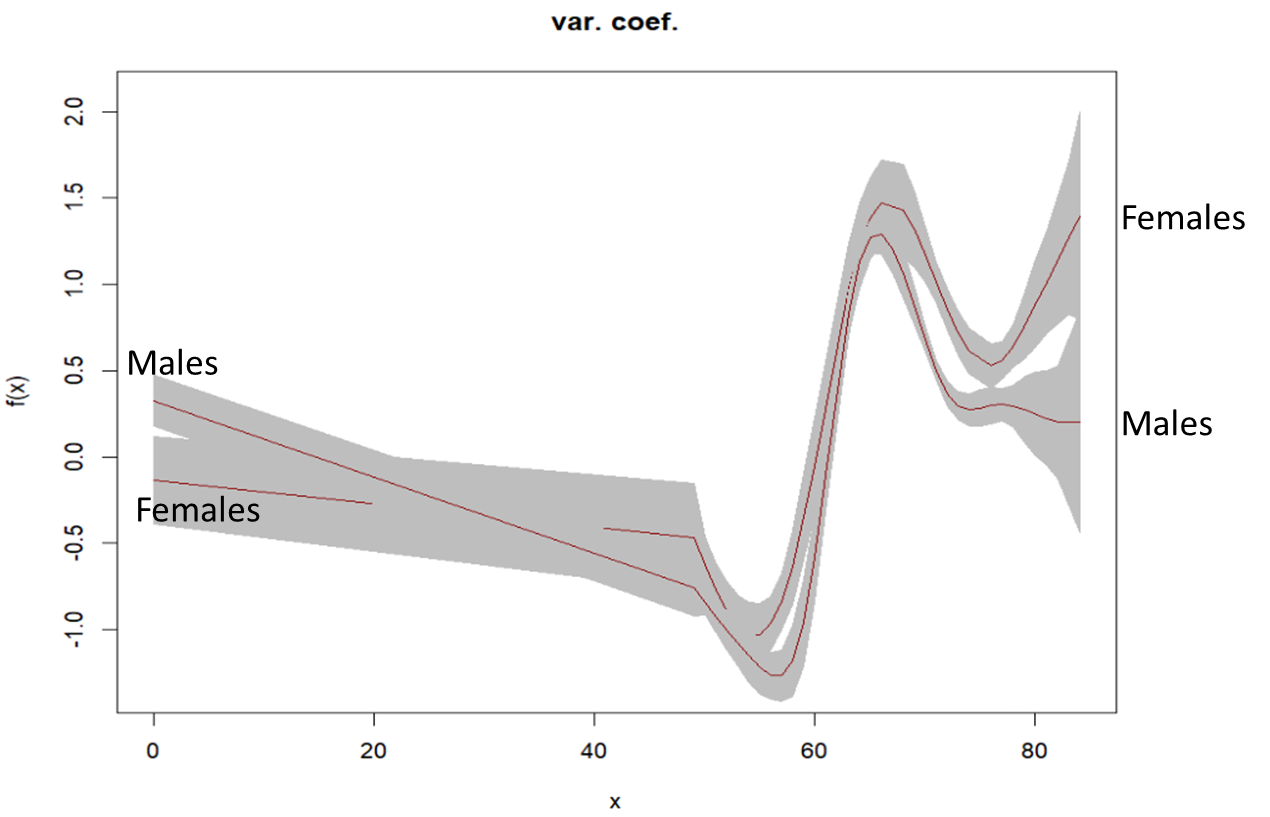


Men started with higher Ebola virus binding antibody geometric concentrations at baseline than women, a boost in antibody response was observed in both men and women from Day 57 until Day 70, with women reaching a higher antibody response than men from Day 70 onwards.
